# Impact of expansive catastrophic plan eligibility on 2026 Marketplace enrollment

**DOI:** 10.1093/haschl/qxag142

**Published:** 2026-06-08

**Authors:** David M Anderson, Dylan Nagy, Coleman Drake

**Affiliations:** Department of Health Services Policy and Management, University of South Carolina, Columbia, SC 29208, United States; Department of Health Policy and Management, University of Pittsburgh, Pittsburgh, PA 15261, United States; Department of Health Policy and Management, University of Pittsburgh, Pittsburgh, PA 15261, United States

**Keywords:** Affordable Care Act, health insurance, health insurance marketplaces, public policy

## Introduction

In September 2025, the Centers for Medicare and Medicaid Services (CMS) issued new guidance that substantially increased the number of people eligible to purchase catastrophic-level coverage in the Health Insurance Marketplaces.^1^ Historically, catastrophic plans were only available to enrollees below age 30 years or who met hardship exemptions.^2^ Beginning in 2026, CMS expanded catastrophic coverage eligibility to enrollees with incomes above 250% of the federal poverty level (FPL); hardship exemptions were also simplified.^1^

Catastrophic plans have deductibles equal to their maximum out-of-pocket limits: $10 600 for an individual or $21 200 for a family. Except for limited primary care, catastrophic plans leave enrollees fully exposed to medical expenses until they reach their out-of-pocket limit. Catastrophic plans typically have lower premiums than Bronze plans. Bronze plans may have similar or lower out-of-pocket limits and deductibles as well as selected services carved out from cost-sharing—for instance, the lowest premium Bronze plan in Columbia, South Carolina, has a $10 600 deductible and out-of-pocket maximum limit. Unlike Bronze plans, however, catastrophic plans are not eligible for premium subsidies. Subsidies make Bronze plans more affordable for subsidy-eligible enrollees (ie, <400% FPL in 2026), although catastrophic plans’ premiums are more affordable for subsidy-ineligible enrollees.^3^ Marketplace insurers are not required to offer catastrophic plans.^3^

It remains unclear whether catastrophic plan eligibility expansion increased on Marketplace enrollment in 2026. Although the change provided some enrollees with access to lower premium plans, the enrollment effects of these premium decreases may have been muted: subsidy-eligible enrollees already had access to affordable Bronze coverage and enrollees may be averse to catastrophic plans’ less generous coverage.

## Methods

We used a difference-in-differences approach to estimate the impact of expanded catastrophic plan eligibility on enrollment, leveraging the fact that expansion could only affect counties where catastrophic plans were available. We compared 952 counties where catastrophic plans were always available with 244 counties where they were never available, before and after expanded catastrophic eligibility. Counties with intermittent catastrophic availability were excluded due to endogeneity concerns. Our sample frame was counties in states that used HealthCare.gov for all years from 2022 to 2026. Our final sample included 5980 county-years in 21 states. Enrollment data came from the CMS Open Enrollment Period Public Use File; plan offerings and premiums came from HealthCare.gov's Qualified Health Plan Landscape Files. Enrollment data are by year-county-income cell.

We examined enrollment for Marketplace enrollees at or above 250% FPL, the minimum income threshold for catastrophic eligibility in 2026. We separately estimated enrollment by income group, from 250%-300%, 300%-400%, and over 400% FPL. We controlled for the minimum cost of coverage to purchase Marketplace coverage outside of catastrophic coverage—measured as the lowest monthly cost of postsubsidy Bronze coverage—to capture the counterfactual minimum premium per prior work on this measure.^3^ We included state-by-year fixed effects to capture state-level policy and market changes (eg, Medicaid expansion) and county fixed effects to capture time-invariant county health, demographic, and insurance market characteristics. To further capture differences between intervention and control counties, we used entropy balancing to weight-control counties to match the 2025 age and income distributions of Marketplace enrollees in intervention counties, immediately preceding catastrophic coverage eligibility expansion.

We estimated log-linear models to account for the skewed distribution of enrollment and clustered SEs by county. Causal identification relies on the parallel trends assumption, which requires that enrollment would have evolved similarly in counties where catastrophic plans were and were not offered if CMS had not expanded catastrophic coverage eligibility. We support this assumption with pretrends tests.

## Results

Expanded catastrophic coverage eligibility was not associated with significant enrollment changes among Marketplace enrollees with incomes from 250%-300% or 300%-400% FPL. Point estimates are negative for both groups, and the upper bounds of the 95% CIs rule out enrollment increases larger than 1.9% among 250%-300% FPL enrollees and 5.8% among 300%-400% FPL enrollees. Expanded catastrophic coverage eligibility was associated with an 18.3% (95% CI: 2.8% to 36.3%) increase in enrollment among enrollees with incomes above 400% FPL. Pretrends were stable for all outcomes. See Table 1 for details.

**Table 1.** Event study difference-in-differences estimates of catastrophic coverage eligibility expansion's impact on HealthCare.gov enrollment by income group, 2022–2026.

	Income group by percentage of FPL, estimates [95% CI]		
	250%–300% FPL	300%–400% FPL	>400% FPL
Estimates: years to treatment			
−4 (2022)	−10.6 [−20.2, 0.2]	12.7* [2.2, 24.4]	−0.8 [−17.7, 19.6]
−3 (2023)	−12.5 [−24.8, 1.9]	5.9 [−3.1, 15.7]	12.3 [−7.3, 35.9]
−2 (2024)	−3.1 [−12, 6.8]	1.5 [−4.1, 7.3]	9.1 [−13.5, 37.6]
−1 (2025)	0 (ref)	0 (ref)	0 (ref)
0 (2026—Implementation)	−7.7 [−16.4, 1.9]	−0.4 [−6.2, 5.8]	18.3* [2.8, 36.3]
Fixed effects			
County	X	X	X
State-by-year	X	X	X
Model statistics			
Pretrends test	*F* = 2.09 (*P* = .099)	*F* = 1.97 (*P* = .116)	*F* = 0.95 (*P* = .416)
No.	5970	5971	5932

Abbreviations: FPL = Federal Poverty Level as a measure of income; REF= Reference Year.

Estimates were obtained from log-linear models of Marketplace enrollment in 21 states that used HealthCare.gov for all years from 2022 to 2026. Estimates were retransformed from log-linear estimates and SEs were calculated using the delta method. The unit of analysis was the county-year. The sample was restricted to counties that offered or did not offer catastrophic plans consistently from 2022 to 2026 (ie, counties where they were intermittently available were excluded). Standard errors were clustered by county, the unit at which availability of catastrophic coverage varies. Counties were weighted using entropy weights, which were calculated using baseline (2025) values for the age and income distributions of Marketplace enrollees. 2025 Enrollment was used as the base weight when calculating entropy weights. X indicates that a fixed effect was used in for this characteristic in this specification. *P < .05, **P < .01, ***P < .001.

## Discussion

In this difference-in-differences analysis, we found preliminary evidence that catastrophic coverage eligibility expansion was not associated with increases in Marketplace enrollment for enrollees with incomes from 250% to 400% FPL, who typically receive premium subsidies. This is consistent with prior research that found catastrophic coverage eligibility expansion did not improve premium affordability for subsidized Marketplace enrollees.^3^

We did find, however, that catastrophic coverage eligibility expansion increased HealthCare.gov-based coverage by 18.3% among enrollees with incomes over 400% FPL in the counties that always offered catastrophic plans relative to those that did not. In absolute terms, this effect is modest: total nationwide catastrophic enrollment was approximately 67 000 in 2026,^5^ so the additional enrollment of those with incomes greater than 400% FPL attributable to eligibility expansion is, at most, a fraction of that figure. This enrollment gain occurred amidst an overall decline in over-400% FPL enrollment driven by the expiration of the Inflation Reduction Act–enhanced premium tax credits. In our 5980-county, 21-state sample, enrollment above 400% FPL declined from 472 020 in 2025 to 305 602 in 2026. Our findings indicate that enrollment declined less in counties where catastrophic plans were always available; our prior research indicates that this smaller decline is attributable to catastrophic coverage eligibility expansion lowering the minimum cost of Marketplace coverage by approximately $100 per month for a single, 40-year-old adult in affected counties.^3^

We note several limitations. First, our sample does not include states operating state-based Marketplaces. Second, we cannot determine to what extent changes in enrollment are driven by new enrollment vs plan switching as the enrollment data are limited. Third, we did not observe off-Marketplace enrollment; thus, our over-400% FPL estimates are minimum lower bounds as these enrollees are ineligible to receive premium subsidies and have no motivation to use HealthCare.gov. Fourth, we are unable to provide demographic information due to the structure of the publicly available data. Our estimates must be taken with caution as a single post-period limits the strength of the causal identification.

This study provides limited quasi-experimental evidence that catastrophic coverage eligibility expansion did not meaningfully offset coverage losses from the expiration of the Inflation Reduction Act–expanded premium subsidies. The Congressional Budget Office expects 4 million people will lose coverage in 2026; this dwarfs total catastrophic enrollment by nearly 2 orders of magnitude.^4,5^ Reversing these coverage losses will require US Congress or states to act.

## Supplementary Material

qxag142_Supplementary_Data

## Data Availability

All data is publicly available from the Centers for Medicare and Medicaid Services and free to use.
